# Diagnostic accuracy of first and early second trimester multiple biomarkers for prediction of gestational diabetes mellitus: a multivariate longitudinal approach

**DOI:** 10.1186/s12884-021-04348-6

**Published:** 2022-01-04

**Authors:** Elham Shaarbaf Eidgahi, Malihe Nasiri, Nourossadat Kariman, Nastaran Safavi Ardebili, Masoud Salehi, Maryam Kazemi, Farid Zayeri

**Affiliations:** 1 Department of Biostatistics, School of Allied Medical Sciences, Shahid Beheshti University of Medical Sciences, Tehran, Iran; 2 Department of Basic Sciences, Faculty of Nursing and Midwifery, Shahid Beheshti University of Medical Sciences, Tehran, Iran; 3 Department of Midwifery and Reproductive Health Research Center, Faculty of Nursing and Midwifery, Shahid Beheshti University of Medical Sciences, Tehran, Iran; 4 Department of Midwifery, Ardabil Branch, Islamic Azad University, Ardabil, Iran; 5 Health Management and Economics Research Center and Department of Biostatistics, School of Public Health, Iran University of Medical Sciences, Tehran, Iran; 6 Proteomics Research Center and Department of Biostatistics, School of Allied Medical Sciences, Shahid Beheshti University of Medical Sciences, Qods Square, Darband Street, Tehran, Iran

**Keywords:** Gestational diabetes mellitus, Hemoglobin, Hematocrit, Fasting blood sugar, Red blood cell count, Diagnostic accuracy

## Abstract

**Background:**

Gestational Diabetes Mellitus (GDM) is an underlying cause of maternal and newborn morbidity and mortality all around the world. Timely diagnosis of GDM plays an important role in reducing its adverse consequences and burden. This study aimed to determine diagnostic accuracy of multiple indicators in complete blood count (CBC) test for early prediction of GDM.

**Methods:**

In this prospective cohort study, the data from 600 pregnant women was analyzed. In the study sample, the two-step approach was utilized for the diagnosis of GDM at 24–28 weeks of gestation. We also used the repeated measures of hemoglobin (Hb), hematocrit (Hct), fasting blood sugar (FBS) and red blood cell count (RBC) in the first and early second trimesters of pregnancy as the longitudinal multiple indicators for early diagnosis of GDM. The classification of pregnant women to GDM and non-GDM groups was performed using a statistical technique based on the random-effects modeling framework.

**Results:**

Among the sample, 49 women (8.2%) were diagnosed with GDM. In the first and early second trimester of pregnancy, the mean HcT, Hb and FBS of women with GDM was significantly higher than non-GDMs (*P* < 0.001). The concurrent use of multiple longitudinal data from HcT, Hb, RBC and FBS in the first and early second trimester of pregnancy resulted in a sensitivity, specificity and area under the curve (AUC) of 87%, 70% and 83%, respectively, for early prediction of GDM.

**Conclusions:**

In general, our findings showed that the concurrent use of repeated measures data on Hct, Hb, FBS and RBC in the first and early second trimester of pregnancy might be utilized as an acceptable tool to predict GDM earlier in pregnancy.

## Introduction

Gestational Diabetes Mellitus (GDM) is one of the most common medical complications of pregnancy with considerable increasing prevalence in some parts of the world within the previous decades, especially in developing countries [1–3]. It mainly refers to any degree of glucose intolerance with onset or first diagnosis during pregnancy [1, 4]. Although GDM is mostly symptom-free, it might be an underlying cause of acute or long-term consequences both in mothers and babies. Compared to non-GDM women, mothers with GDM have an increased risk of pre-eclampsia, mental disorders like depression, urinary tract infections, type II diabetes, and cardiovascular disease [5–8]. In addition, untreated GDM may result in some health problems in newborns such as macrosomia, having low blood sugar, and icterus. Later in life, babies born from GDM women are at risk of being overweight or obese and developing type II diabetes [5, 9, 10].

Although OGTT is the globally accepted diagnostic criteria for GDM, the reference values used in different countries might be dissimilar. Depending on the diagnostic criteria, researchers have reported a wide range of prevalence rates for GDM in different parts of the world. The Middle East and North Africa had the highest and Europe had the lowest prevalence rate of GDM in recent years (with the estimated median of 12.9% and 5.8%, respectively) [11]. The International Diabetes Federation (IDF) has estimated a global prevalence of 14% for GDM in 2013 [12]. The estimates also show that GDM imposes a huge economic burden on the health systems of the world countries [13, 14].

To date, a variety of screening and diagnostic procedures have been utilized for detecting mothers with GDM and these procedures may vary by country, region, and year. For instance, the American Diabetes Association (ADA) and the World Health Organization (WHO) have recommended different criteria for the diagnosis of GDM in pregnant women [15–18]. Apparently, these criteria may lead to different diagnostic power indices (sensitivity, specificity, positive and negative predictive values, area under the ROC curve) subject to the characteristics of the target population [11, 19]. In addition to the standard methods of screening, a number of studies evaluated the diagnostic accuracy of the first or second-trimester maternal markers for early detection of GDM using different statistical techniques [20–27].

Reviewing the related published literature indicates that most researchers have investigated the information from a single biomarker or multiple biomarkers in the first or early second trimester of pregnancy for early detection of GDM. The beta-human chorionic gonadotropin (β-hCG), tumor-necrosis factor alpha (TNFα), unconjugated estriol (uE3), alfa-fetoprotein (AFP), pregnancy-associated plasma protein A (PAPP-A), soluble endoglin, placental protein 13 (PP13), adiponectin (Adipo), and a number of other adipose tissue-derived and placenta-related factors are among these biomarkers [21–25]. At the same time, a number of studies have focused on the available data from the more common indicators in complete blood count (CBC) tests for early prediction of GDM [26, 27].

To the best of our knowledge, all of the published research has used cross-sectional data on a single biomarker or multiple biomarkers in the first or second trimester of pregnancy for early detection of GDM. Regarding this, the main goal of the present study was to predict GDM using multiple repeated measures (multivariate longitudinal) data from four routine indicators in the CBC test in the first and early second trimesters of pregnancy. To do this, two longitudinal discrimination approaches (univariate and multivariate) have been used and the predictive power of these methods in early diagnosis of GDM has been evaluated using specificity, sensitivity, and area under roc curve.

## Methods

### Sample under study

In this prospective cohort study, 700 pregnant women consecutively referred to the prenatal clinic of Milad Hospital in Tehran, Capital of Iran, were recruited. The study protocol was approved by the Ethics Committee of Shahid Beheshti University of Medical Sciences and written informed consent was obtained from all women. At the first step, demographic information and midwifery history of these women were collected by face-to-face interview and the mothers' weight and height was measured using a standard scale and a meter attached to the scale.

The inclusion criteria were singleton pregnancy, gestational age of 13 weeks or less (using a sonography report during the first trimester), maternal age between 18 and 35 years, parity of 3 or less, lack of any diagnosed systemic diseases (including diabetes, chronic hypertension, and cardiovascular disease, chronic renal disease, gastroenterology disease, thyroid, epilepsy, hemoglobinopathies, and mental disorders), lack of any history of smoking, alcohol use, non-routine drugs use during the present pregnancy, and any history of preeclampsia in previous pregnancy. In addition, presence of fetal anomalies, polyhydramnios, oligohydramnios, placenta praevia, abruption placenta, abortion or threatened abortion, premature rupture of membranes (PPROM), stillbirth and use of Assisted Reproductive Technology (ART) were considered as exclusion criteria. Regarding these criteria, 100 subjects were excluded from the study.

### Main outcome and biomarkers under the study

In this research, the main outcome under study was the presence of GDM at 24–28 weeks of gestation. The two-step approach was used for the diagnosis of GDM in the described sample. In the first step of this approach, we performed a 50-g glucose load test (GLT) and measured the plasma glucose at 1 h after test. In the second step, we performed a 100-g oral glucose tolerance test (OGTT) for women with the plasma glucose level of 140 mg/dL or more measured in the first step. Finally, the diagnosis of GDM was made according to the Carpenter and Coustan (C&C) criteria when at least two of the following four plasma glucose levels were met; fasting level of at least 95 mg/dl, 1 h level of at least 180 mg/dl, 2 h level of at least 155 mg/dl and 3 h level of at least 140 mg/dl.

For 600 pregnant women under study, the blood samples were collected repeatedly in the first trimester (gestation age of 12 weeks or less) and early second trimester (during weeks 16–20 of gestation). In this study, we used the data from two repeated measures of hemoglobin (Hb), hematocrit (Hct), fasting blood sugar (FBS), and red blood cell count (RBC) in the described trimesters as the early predictors of GDM.

### Statistical analysis

In the first stage of data analysis, to compare the different characteristics of the women with and without GDM, we used the chi-square test and independent samples t-test. We utilized the repeated measures analysis of variance to compare the repeated levels of the described biomarkers in two trimesters between GDM and non-GDM groups. To achieve the main goal of the present study (evaluating the predictive power of longitudinal multiple biomarkers for early detection of GDM), we applied the method suggested by Roy which uses multivariate longitudinal data (biomarkers) for classifying sampling units into different subgroups (GDM or non-GDM) [28]. In this context, we first use the following multivariate longitudinal random-effects model to assess the effect of different covariates on repeated biomarkers:

$${{\varvec{y}}}_{i}={{\varvec{X}}}_{i}{\varvec{\beta}}+{{\varvec{Z}}}_{i}{{\varvec{b}}}_{i}+{{\varvec{\varepsilon}}}_{i}$$

$${{\varvec{b}}}_{i}\sim N\left(0, {\varvec{D}}\right)$$

$${{\varvec{\varepsilon}}}_{i}\sim N\left(0, {\boldsymbol{\Omega }}_{{\varvec{i}}}\right)$$

where ***y***_*i*_, ***X***_*i*_, ***β***, ***b***_*i*_, ***Z***_*i*_ and ***ε***_***i***_ indicate the response variables, matrix of time-stationary (such as parity and mother educational level) and time-varying (such as Body Mass Index, time of measurement, systolic and diastolic blood pressure) covariates, vector of regression parameters, matrix of time-varying covariates, random term, and random errors, respectively. In this model, ***D*** and ${{\varvec{\Omega}}}_{{\varvec{i}}}$ show the variance–covariance matrices of random terms and random errors, respectively.

In the modeling process, we considered four repeated biomarkers as the multivariate longitudinal response variables (***y***_i_). In modeling process, maternal age, body mass index (BMI), systolic blood pressure (SBP), diastolic blood pressure (DBP), presence of GDM (1 = non-GDM, 2 = GDM), and time of measurement (1 = first trimester, 2 = second trimester) were considered as the model covariates (***X***_*i*_). After fitting this multivariate longitudinal model and estimating the parameters, we used the following classification rule for discriminating pregnant women in terms of being in non-GDM (population 1) or GDM (population 2) groups:

Allocate the *i*th woman to non-GDM group if

$${{({\varvec{\mu}}}_{1i}-{{\varvec{\mu}}}_{2i})}^{^{\prime}}{\left({{\varvec{Z}}}_{i}{\varvec{D}}{{\varvec{Z}}}_{i}^{^{\prime}}+\mathrm{dim}({\boldsymbol{\Omega }}_{{\varvec{i}}}\right))}^{-1}\left({{\varvec{y}}}_{i}-\frac{1}{2}\left({({\varvec{\mu}}}_{1i}+{{\varvec{\mu}}}_{2i}\right)\right)\ge ln\left(\frac{{{\varvec{\pi}}}_{2}}{{{\varvec{\pi}}}_{1}}\right)$$

and to GDM group otherwise. In this equation, π_1_ and π_2_ indicate the proportion of population 1 and 2 (in our study, the proportion of GDM and non-GDM women), μ_i1_ and μ_i2_ show the mean response variables for *i*th subject in group 1 and 2 (in our study, the mean values of biomarkers in GDM and non-GDM women). In addition, notation *dim* indicates the dimension of the matrix.

In this discrimination technique, two different strategies were utilized to assess the power of RBC, Hb, HcT, and FBS for predicting GDM. At the first stage, we used the repeated measures data (biomarker level in the first and second trimesters) from each biomarker to estimate the predictive performance of every single biomarker under the study (univariate strategy). In the next stage, the repeated measures data from all the described biomarkers were used in a multivariate framework to determine the predictive power indices of all biomarkers concurrently (multivariate strategy).

## Results

The mean (SD) age and BMI of the 600 pregnant women was 27.28 (3.94) and 24.99 (4.37), respectively. More than one-fourth of women under the study had academic education.

Among the sample pregnant women, 49 cases (8.2%) were diagnosed with GDM by the end of trimester 3. Table 1 shows the characteristics of these women by GDM. As we can see, the mean age of women with GDM was significantly higher than non-GDMs (*P* = 0.048). In the second trimester of pregnancy, the mean systolic and diastolic blood pressures were also significantly higher in GDM patients than non-GDM women (*P* = 0.005 and *P* = 0.008, respectively).

**Table 1:** Comparing the demographic and clinical characteristics between two groups

**Characteristic**	**Category**	**Non-GDM(*n* = 551)**	**GDM(*n* = 49)**	**p**
Parity	**0**	1(0.2)^a^	0(0)	0.689
	**1**	260(47.2)	28(57.1)	
	**2**	224(40.6)	15(30.7)	
	**3**	66(12)	6(12.2)	
Education	**Non-Academic**	399(72.4)	38(77.6)	0.816
	**Academic**	152(27.6)	11(22.4)	
Folic Acid use	**Yes**	530(96.2)	47(95.9)	0.925
	**No**	21(3.8)	2(4.1)	
Iron use	**Yes**	539(97.8)	49(100)	0.356
	**No**	12(2.2)	0(0)	
Calcium use	**Yes**	366(66.4)	33(67.3)	0.896
	**No**	185(33.6)	16(32.7)	
Vitamin use	**Yes**	432(78.4)	42(85.7)	0.229
	**No**	119(21.6)	7(14.3)	
Age (years)	**-**	27.19 ± 3.93^b^	28.35 ± 3.89	0.048
BMI (trimester 1)	**-**	24.89 ± 4.30	26.09 ± 5.02	0.066
BMI (trimester 2)	**-**	29.73 ± 4.11	30.90 ± 4.45	0.059
DBP (trimester 1)	**-**	64.2 ± 7.7	63.47 ± 8.05	0.525
DBP (trimester 2)	**-**	66.16 ± 9.57	70.01 ± 10.60	0.008
SBP (trimester 1)	**-**	101.79 ± 12.31	101.73 ± 10.78	0.977
SBP (trimester 2)	**-**	106.56 ± 14.43	112.55 ± 13.62	0.005

^a^No(%)

^b^$$\overline{\mathrm{x}}\pm \mathrm{SD }$$

Table 2 shows the results from the repeated measures analysis of variance test for comparing the mean trend of four biomarkers (RBC, Hb, Hct, and FBS) between women with and without GDM. In this context, we first assessed the interaction between group and time variables. Since none of the interactions were significant, we only reported the group effect in the last column of Table 2 to compare the mean trend of the biomarkers between two groups. From these results, one can conclude that the mean trends of Hb, Hct, and FBS were significantly higher in GDM patients compared to non-GDMs (*P* < 0.001). At the same time, although the mean RBC was slightly higher in GDMs than non-GDM women, there was no significant difference between two groups (*P* = 0.080).

**Table 2:** Comparing the mean trend of different markers in GDM and non-GDM patients

**Marker**	**Trimester**	**GDM**	**Non- GDM**	**P**
**RBC (× 10**^**12**^**)**	1 2	4.58 ± 0.45 4.19 ± 0.41	4.39 ± 5.01 4.12 ± 0.45	0.080
**Hb (g/dL)**	1 2	13.22 ± 1.17 12.32 ± 0.90	12.63 ± 0.99 11.89 ± 0.97	< 0.001
**Hct (%)**	1 2	39.53 ± 3.35 36.87 ± 2.54	37.55 ± 3.54 35.86 ± 2.88	< 0.001
**FBS (mg/dL)**	1 2	92.04 ± 12.01 96.96 ± 12.74	83.19 ± 11.75 81.08 ± 10.17	< 0.001

**Fig. 1:**
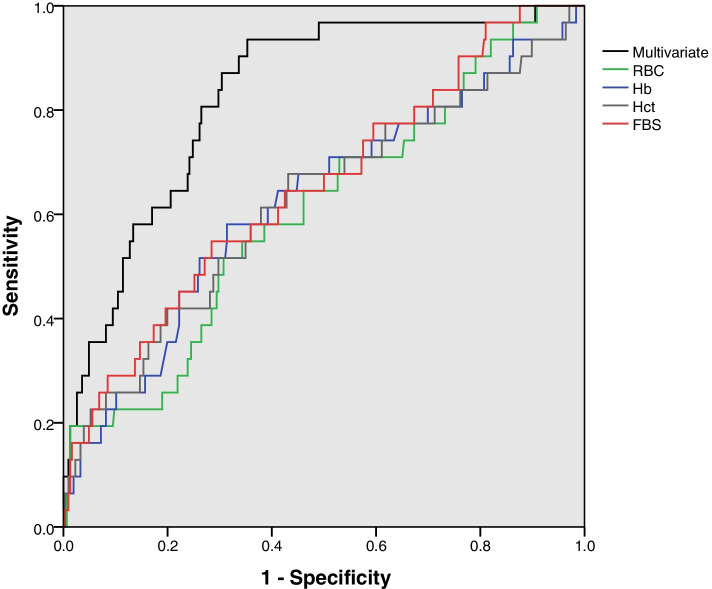
Roc curves for univariate and multivariate analysis of markers

In the next step of data analysis, we used the ordinary univariate ROC curve analysis in addition to the explained multivariate longitudinal discriminant analysis to identify the predictive power of markers in early diagnosis of GDM. The estimated values of sensitivity, specificity, and area under curve for these markers were presented in Table 3. As can be seen, when we use RBC, Hb, Hct, and FBS as a single indicator for predicting GDM (univariate analysis), the estimated values of AUC seem to be quite unsatisfactory (ranging from 61% for RBC to 68% for FBS). On the other hand, when we simultaneously utilized these biomarkers for predicting GDM (multivariate analysis), more acceptable estimates could be obtained for the sensitivity, specificity, and subsequently AUC indices. In other words, concurrent use of these biomarkers for early prediction of GDM would results in an estimated sensitivity of 87%, specificity of 70%, and AUC of 83%. Figure 1 displays the estimated ROC curves from univariate and multivariate analysis of the biomarkers.

**Table 3:** Estimated predictive power indices using univariate and multivariate analysis of different markers in early diagnosis of GDM

**Marker**	**Sensitivity**	**Specificity**	**AUC**	**95%CI for AUC**
RBC	60	54	61	50–71
Hb	67	62	67	61–73
HcT	62	61	65	59–71
FBS	68	60	68	60–75
Multivariate	87	70	83	76–90

## Discussion

In our data analysis process, we first compared the mean repeated measures of RBC, Hb, Hct, and FBS between GDM and non-GDM groups. The results revealed that the mean levels of these four biomarkers were higher in women with GDM than non-GDM (all p-values were significant except for RBC which showed a borderline significancy). The relationship between GDM and some of these biomarkers has been previously evaluated by other researchers in a number of cross-sectional or cohort studies. In a prospective study conducted by Wu et al., they compared some clinical characteristics of women with and without GDM during weeks 12–16 of gestation. Similar to our results, they reported significant higher mean levels of RBC, Hb, Hct, and FBS in women with GDM compared to healthy pregnancies. Their findings also showed higher levels of BMI, SBP, DBP, platelet count, glycated hemoglobin, total cholesterol, triglyceride, HDL, and folic acid as well as lower levels of glacyted albumin and vitamin B12 in GDMs compared to non-GDMs [27]. The findings from a hospital-based retrospective study among more than 21,000 Chinese pregnant women showed a significant relationship between increased Hb levels during early pregnancy and development of GDM. They also reported an odds ratio of 1.27 for developing GDM in women with increased levels of Hb compared to other pregnant women [26]. In another cohort study among 600 Iranian pregnant women, estimated relative risks of 2.12 and 1.47 were reported for developing GDM in women with high Hb levels compared to those with normal Hb, respectively in the second and third trimesters of pregnancy [29]. Regarding these findings, it can be concluded that all of the described biomarkers could be used as potential diagnostic indicators for early detection of GDM.

In the present study, our main objective was to investigate the diagnostic accuracy of some available indicators in routine blood test for early detection of GDM. Our findings showed that using the repeated measures of RBC, Hb, Hct and FBS in the first and early second trimesters of pregnancy leads to a sensitivity of 87%, specificity of 70%, and AUC of 83% for early prediction of GDM. In other words, the estimated AUC tells us that more than four-fifth of pregnant women could be correctly classified into GDM or non-GDM group using the multiple repeated measures of the described biomarkers. As mentioned in the introduction section, a number of studies have previously examined the diagnostic power of different biomarkers for predicting the development of GDM. In a case–control study on 12 GDM patients and 60 controls, the researchers used the logistic regression model and concluded that combination of Adipo, PAPP-A and BMI yields a detection rate of 72.7% at a false positive rate of 10% for detecting GDM [20]. The lower detection rate in comparison with our study might be due to simple univariate statistical methods or inadequate sample size in this research. In another study on 523 pregnant women, Amini et al. applied more complicated statistical modeling approach (Bayesian latent class models) to assess diagnostic accuracy of multiple markers for early detection of GDM. Using the β-hCG, uE3, and AFP as the multiple biomarkers, they reached a sensitivity of 94%, specificity of 86%, and AUC of 92% [21]. Analyzing the data using a Bayesian approach in this study can be considered as an advantage because this statistical methodology usually leads to more reliable estimates when the sample size is small. In another study by Tenenbaum-Gavish et al., the researchers used obesity, placental, and inflammatory biomarkers for prediction of GDM in the first trimester of pregnancy. Their results showed an AUC of 95% with detection rate of 89% at 10% false positive rate for predicting GDM in obese expectant women using the combination of high BMI, insulin, soluble CD163, and TNFα results. They also reported an AUC of 94% with detection rate of 83% at 10% false positive rate for prediction GDM in non-obese women using the combination of soluble CD163, TNFα, PP13, and PAPP-A [22]. Zhao et al. used the data from 876 singleton pregnancies to assess the predictive power of early second trimester maternal serum markers in prediction of GDM. At a specificity level of 80%, their findings showed detection rate of 94.9% for pentraxin 3 (PTX3), 92.3% for PP13, 94.9% for soluble fms-like tyrosine kinase-1 (sFLt-1), 92.5% for myostatin and 92.3% for follistatin (FST) [24]. Wu et al. evaluated the accuracy power of glycated hemoglobin A1c (HbA1c) alone and in combination with HcT for screening GDM patients between 12–16 gestational weeks. They concluded that HbA1c as a single biomarker had no acceptable predictive power for detecting GDM (with an AUC of 56.3%) while the combination of HbA1c and HcT might be a useful screening test for GDM (with an AUC of 60.4%) [27]. In a recent research in this field, Lorenzo-Almorós et al. reviewed large number of predictive and diagnostic biomarkers for GDM including protein and genetic biomarkers they concluded that reduction in liver-derived sex hormone blinding globulin (SHBG) and adiponectin, in combination with increased levels of RBP4, afamin, ficlon-3, and some specific MiRs could be a reliable measure for prediction GDM [26]. As can be seen, higher predictive values have been reported for early detection of GDM in some of the above mentioned studies using different biomarkers. However, using highly available inexpensive markers in addition to applying longitudinal data and more sophisticated statistical methods which gives us valuable information about the trends of change in these markers, might be considered as the main advantages of our reaerch in comparison with other studies in this field.

In conclusion, reviewing the published works in this field indicates that there is no consensus among researchers regarding the best feasible framework for early prediction of GDM. It seems that more comprehensive studies with higher sample sizes and wider range of biomarkers should be conducted to identify more reliable and globally practicable standard for the diagnosis of GDM in the earlier stages of pregnancy.

Similar to other studies in this field, our study has some limitations and strengths. In our opinion, applying a complex statistical method which allows us to concurrently include the information from the longitudinal multiple biomarkers (Hct, Hb, FBS, and RBC levels in the first and early second trimesters of pregnancy) in a single predictive tool was the most important strength point of the current study. By using this approach, we presented a feasible framework for early prediction of GDM via inexpensive accessible parameters from the routine CBC tests in different trimesters of pregnancy. In addition, analyzing the data from a relatively adequate sample size, at least in comparison with most of the related studies in this field (assessing the predictive power of different biomarkers for early detection of GDM), could be considered as another strength point of the present work. On the other hand, using a non-random sampling technique (consecutive sampling method) for selecting the study sample might be thought as the main limitation of our study. This limitation may call into question the generalization of some inferences in our work. Regarding this, conducting more comprehensive studies using random sampling approaches (for instance, multi-stage cluster sampling technique) and more generalizable sample sizes might be needed to evaluate the predictive power of these biomarkers for early detection of GDM more accurately.

## Conclusion

In this study, we employed more advanced statistical methods to suggest a framework for early detection of GDM by using common parameters in the routine CBC tests. The estimated indices of predictive accuracy for the described method showed that about 80% of pregnant women could be properly categorized into GDM and non-GDM groups using our suggested framework. Although some researchers have previously introduced a number of biomarkers with higher predictive power for early detection of GDM, some of these biomarkers are costly or hard to access for all pregnant women in the majority of poor or low-income countries. Thus, there is an urgent need in the future works to focus on more popular and accessible indicators for early prediction of GDM, especially in countries with lower levels of health resources. In addition, more convincing results about the predictive power of the utilized indicators might be obtained by increasing sample size and conducting multi-center studies in the future.

## Methods and procedures

All the methods and procedures described in the present study, were performed according to the relevant guidelines and regulations following the declaration of Helsinki.

## Author statement

The corresponding author declares that the present study has not been published before in any other journal.

## Data Availability

The dataset analyzed in this study might be available from the corresponding author on reasonable request.

## Abbreviations

GDM: Gestational Diabetes Mellitus
CBC: Complete Blood Count
PPROM: Premature Rupture of Membranes
ART: Assisted Reproductive Technology
Hb: Hemoglobin
Hct: Hematocrit
FBS: Fasting Blood Sugar
RBC: Red Blood Cell Count
AUC: Area Under the Curve
IDF: International Diabetes Federation
ADA: American Diabetes Association
WHO: World Health Organization
β-hCG: Beta-human Chorionic Gonadotropin
TNFα: Tumor-Necrosis Factor Alpha
uE3: Unconjugated Estriol
AFP: Alfa-Fetoprotein
PAPP-A: Pregnancy-Associated Plasma Protein A
PP13: Placental Protein 13
Adipo: Adiponectin
GLT: Glucose Load Test
OGTT: Oral Glucose Tolerance Test
BMI: Body Mass Index
SBP: Systolic Blood Pressure
PTX3: Pentraxin 3
sFLt-1: Soluble Fms-Like Tyrosine Kinase-1
FST: Follistatin
HbA1c: Glycated Hemoglobin A1c
SHBG: Sex Hormone Blinding Globulin
